# Trends in antenatal care visits and associated factors in Ghana from 2006 to 2018

**DOI:** 10.1186/s12884-022-04404-9

**Published:** 2022-01-22

**Authors:** Precious Adade Duodu, Jonathan Bayuo, Josephine Aboagye Mensah, Livingstone Aduse-Poku, Francis Arthur-Holmes, Veronica Millicent Dzomeku, Nutifafa Eugene Yaw Dey, Pascal Agbadi, Jerry John Nutor

**Affiliations:** 1 Department of Nursing and Midwifery, School of Human and Health Sciences, University of Huddersfield, Queensgate, Huddersfield, England UK; 2 School of Nursing, The Hong Kong Polytechnic University, Kowloon, Hong Kong; 3 Child Health Directorate, Komfo Anokye Teaching Hospital, Post Office Box 1934, Adum -, Kumasi, Ghana; 4 Department of Epidemiology, College of Public Health & Health Professions, College of Medicine, University of Florida, Gainesville, USA; 5 Department of Sociology and Social Policy, Lingnan University, 8 Castle Peak Road, Tuen Mun, Hong Kong; 6 Department of Nursing, Faculty of Allied Health Sciences, College of Health Sciences, Kwame Nkrumah University of Science and Technology, Kumasi, Ghana; 7 Department of Psychology, University of Ghana, P.O. Box LG 84, Legon, Ghana; 8 Department of Family Health Care Nursing, School of Nursing, University of California San Francisco, San Francisco, California, USA

**Keywords:** Prenatal care, maternal and child health, sub-Saharan Africa, child morality, maternal mortality

## Abstract

**Introduction:**

Given that maternal mortality is a major global health concern, multiple measures including antenatal care visits have been promoted by the global community. However, most pregnant women in Ghana and other sub-Saharan African countries do not attain the recommended timelines, in addition to a slower progress towards meeting the required minimum of eight visits stipulated by the World Health Organization. Therefore, this study explored the trends in antenatal care visits and the associated factors in Ghana from 2006 to 2018 using the Multiple Indicator Cluster Surveys.

**Methods:**

The study used women datasets (*N* = 7795) aged 15 to 49 years from three waves (2006, 2011, and 2017-2018) of the Ghana Multiple Indicator Cluster Surveys (GMICS). STATA version 14 was used for data analyses. Univariable analyses, bivariable analyses with chi-square test of independence, and multivariable analyses with robust multinomial logistic regression models were fitted.

**Results:**

The study found a consistent increase in the proportion of women having adequate and optimal antenatal attendance from 2006 to 2018 across the women’s sociodemographic segments. For instance, the proportion of mothers achieving adequate antenatal care (4 to 7 antenatal care visits) increased from 49.3% in 2006 to 49.98% in 2011 to 58.61% in 2017-2018. In the multivariable model, women with upward attainment of formal education, health insurance coverage, increasing household wealth, and residing in the Upper East Region were consistently associated with a higher likelihood of adequate and/or optimal antenatal care attendance from 2006 to 2018.

**Conclusion:**

Women who are less likely to achieve optimal antenatal care visits should be targeted by policies towards reducing maternal mortalities and other birth complications. Poverty-reduction policies, promoting maternal and girl-child education, improving general livelihood in rural settings, expanding health insurance coverage and infrastructural access, harnessing community-level structures, and innovative measures such as telehealth and telemedicine are required to increase antenatal care utilization.

**Supplementary Information:**

The online version contains supplementary material available at 10.1186/s12884-022-04404-9.

## Background

Globally, maternal mortality remains a major public health issue with an estimated 810 women dying from pregnancy-related complications daily [1]. In 2017, the World Health Organization (WHO) recorded approximately 295,000 maternal deaths following pregnancy and childbirth: 94% of these deaths occurred in low-and-middle-income settings with sub-Saharan African (SSA) and Southern Asia accounting for 86% [2]. These have led to calls for more actions to curb the situation as highlighted in the Sustainable Development Goal (SDG) three, which targets a reduction in the global maternal mortality ratio to less than 70 per 100,000 live births by 2030 [3, 4]. Among the strategies to overcome this challenge is timely utilization of antenatal care which remains paramount particularly in SSA [5, 6].

Antenatal care (ANC) refers to the routine care delivered to expectant mothers following conception to the onset of labour [7]. Adequate ANC support offers an opportunity to deliver health promotion and preventive services [8]. Overall, ANC seeks to promote and protect the health of the expectant mother and the unborn baby to improve health outcomes and transition to the post-natal period with minimum challenges. Thus, all pregnant women need to receive adequate and timely ANC support to promote a positive experience during the period of conception. The timing of the first ANC visit is of utmost importance as it helps to plan subsequent visits [9]. As suggested by the previous WHO Focused Antenatal Care (FANC) Framework, an expectant mother should have at least four ANC visits throughout pregnancy [10]. However, the updated framework by WHO in 2016 highlighted a minimum of eight contacts with ANC services to adequately prepare for the delivery process and avoid complications [4, 11–13]. Also, the updated framework emphasised the need for comprehensive and person-centred care at each visit [13]. Despite these new recommendations, approximately 69% of pregnant women in SSA countries have at least only one ANC visit which may suggest that most expectant mothers do not attain the recommended timelines. The report shows that there is much slower progress towards meeting the required minimum number of ANC visits stipulated by WHO in most SSA countries [14].

In Ghana, the national coverage of ANC service for the previously recommended four visits is above the global average [15]. However, rural-urban and regional discrepancies regarding service delivery and utilization may exist as some studies have noted that some expectant mothers, particularly those in the rural settings are unable to access ANC services [16, 17]. The existing evidence so far suggests that regardless of the socio-economic and demographic factors, pregnant women enrolled on the National Health Insurance Scheme (NHIS) are likely to utilise ANC services than those who are not enrolled [18]. Also, pregnant women with formal education, residing in urban areas in Ghana and who are wealthy are more likely to utilize ANC visits than those with no formal education, from poorer households, and those in rural areas [19]. Additionally, access to transportation and number of children influence the level of ANC service utilization [20]. Although these findings help to understand the factors associated with ANC service utilization at a national level, they do not necessarily offer insights into the trends across the regions of Ghana and whether the aforementioned factors may differ on regional basis. It is believed that by exploring existing datasets, it will be possible to uncover regional trends of ANC utilization and its associated factors which can better inform policy and practice. Therefore, this study explored the trends in ANC visits and the consistency in the associated factors associated with ANC visits in Ghana from 2006 to 2018 using the Multiple Indicator Cluster Surveys.

## Methods

### Data source and collection procedure

Women datasets from three waves of the Ghana Multiple Indicator Cluster Survey (GMICS) conducted in 2006, 2011 and 2017-2018 were analyzed for this study. The GMICS is a cross-sectional survey conducted by the Ghana Statistical Service (GSS) in association with the Ghana Health Service (GHS), Ministry of Health (MOH), and the Ministry of Education [21]. Funding and technical support were provided by the United Nations International Children’s Emergency Fund (UNICEF) and other international donors [21]. The main aim of the MICS surveys is to collect data on key indicators that assist countries to produce evidence for use in national development plans, policies, and programmes as well as assess the advancements towards the Sustainable Development Goals (SDGs) and other internationally-signed agreements [21].

Trained research enumerators were engaged to collect the data on behalf of GSS and UNICEF using a multi-stage stratified cluster sampling approach. This approach nationally surveyed women in urban and rural areas from the previous ten administrative regions in Ghana: Western, Central, Greater Accra, Volta, Eastern, Ashanti, Brong Ahafo, Northern, Upper East, and Upper West. The initial stage of data collection involved identifying and selecting enumeration areas based on the 2010 Population and Housing Census of Ghana. These enumeration areas became the primary sampling units. Next, in the second stage, households were listed from each of the selected enumeration areas and a sample of households was selected using systematic random sampling. This stage enabled the recruitment of reproductive-aged women from selected households. Data of 7795 women aged 15 to 49 years from all the three waves who had delivered 2 years prior to the data collection periods were included in this study.

### Measures

#### Outcome variable

Antenatal care attendance is the main outcome variable for this study. This variable was extracted from a single-item survey question asking women who had given birth 2 years prior to the data collection about the number of times they attended antenatal care. Women were specifically asked, “How many times did you receive antenatal care during this pregnancy?” Women responded by providing a single number or range of numbers. For those who responded by giving a range, the minimum number was recorded as their answer. Guided by the WHO’s recommendation, these numbers were categorized under 4-scale response format: “none = 1”, “1-3 visits = 2”, “4+ visits = 3” and 8+ visits = 4″. We decided to collapse the none and “1-3 visits” into one category as “less than 4 visits” because only one woman did not attend ANC in the 2006 data. This categorization makes it easy for us to compare the models for the three data waves. Therefore, the newly created categories are as follows: “less than 4 visits (undesirable)=0”, “4 to 7 visits (adequate)=1” and 8+ visits (optimal) = 2″.

#### Explanatory variables

Age of woman, education, polygyny status, wanted last-child, parity, death of a previous child, health insurance, household wealth index, urban-rural residence, and region of residence were treated as explanatory variables as seen in Table 1. We selected the variables from the datasets based on their reported significance to the outcome variable in the literature [5, 22, 23]. All variables were available in all the three datasets except for health insurance which was only available in the 2011 and 2017-2018 datasets. The variables were measured with single-item self-report questions and simple categorical response options. For instance, age of woman was measured with the question, “How old are you?” and participants responded by indicating their age in numbers which was later categorized by UNICEF. Health insurance was measured with the question, “Are you covered by any health insurance?” with response format comprising “Yes = 1” and “No = 2”. Education was measured by asking participants to respond to the question, “What is the highest level and grade or year of school you have attended?” with responses ranging from, “early childhood education=0” to “higher = 6”. We used the Variance Inflation Factor (VIF) to check for the assumptions of multicollinearity among the independent variables, and we have not observed any violations.

**Table 1:** Cross-tabulation between ANC visits and study variables in Ghana from 2006 to 2017-2018

	2006				2011				2017-2018			
	n (%)	< 4	4-7	8+	n (%)	< 4	4-7	8+	n (%)	< 4	4-7	8+
Total	1456 (100)	389 (26.7)	717 (49.2)	350 (24.1)	2873 (100)	384 (13.4)	1436 (50.0)	1053 (36.6)	3466 (100)	519 (15.0)	2031 (58.6)	915 (26.4)
**Age (years)**	***P*** **≤ 0.05**				***P*** **≤ 0.005**				***P*** **≤ 0.001**			
15-24	433 (29.7)	31.4	49.6	19.0	705 (24.5)	17.1	50.8	32.1	952 (27.5)	17.5	60.4	22.1
25-34	693 (47.6)	23.8	48.7	27.5	1436 (50.0)	10.6	48.0	41.4	1634 (47.2)	13.5	56.8	29.7
35+	330 (22.7)	26.6	49.8	23.6	733 (25.5)	15.2	53.1	31.7	879 (25.4)	14.9	60.1	25.0
**Education level**	***P* ≤ 0.001**				***P* ≤ 0.001**				***P*** **≤ 0.001**			
None or pre-primary	537 (36.9)	35.0	48.9	16.1	833 (29.0)	22.1	59.5	18.4	774 (22.3)	20.1	64.2	15.8
Primary	320 (22.0)	34.0	48.2	17.8	642 (22.3)	15.3	50.3	34.4	729 (21.0)	21.1	57.4	21.5
JHS	496 (34.1)	17.2	52.6	30.3	1007 (35.0)	9.0	47.2	43.8	1341 (38.7)	12.3	61.2	26.5
Secondary & above	103 (7.1)	6.8	38.1	55.1	391 (13.6)	2.9	37.0	60.9	622 (17.9)	7.3	47.5	45.2
**Polygyny**	***P*** **≤ 0.05**				***P*** **≤ 0.001**				***P*** **≤ 0.001**			
Never/ formerly married	165 (11.3)	34.5	47.2	18.4	293 (10.2)	15.8	43.8	40.4	592 (17.1)	19.8	55.3	25.0
In one union	1027 (70.5)	25.1	47.8	27.0	2112 (73.5)	10.6	49.7	39.8	2331 (67.3)	13.0	58.0	29.0
Have co-wives	264 (18.1)	28.0	56.0	16.0	468 (16.3)	24.6	55.2	20.2	543 (15.7)	18.2	64.8	17.0
**Wanted the last child**	***P* ≤ 0.001**				***P* ≤ 0.05**				***P* ≤ 0.001**			
Yes	884 (60.7)	20.1	52.1	27.8	1630 (56.7)	11.32	49.4	39.3	1711 (49.37)	12.14	58.57	29.3
Later/No More/others	572 (39.3)	36.9	44.83	18.31	1243 (43.3)	16.1	50.8	33.1	1755 (50.6)	17.8	58.7	23.6
**Parity**	***P*** **≤ 0.05**				***P*** **≤ 0.001**				***P*** **≤ 0.01**			
Primiparous	321 (22.0)	26.5	47.2	26.3	619 (21.5)	7.3	47.7	45.1	791 (22.8)	13.5	54.0	32.5
Double	301 (20.7)	20.6	50.6	21.5	527 (18.3)	13.9	47.4	38.7	660 (19.1)	12.7	58.4	29.0
Multiparous	834 (57.3)	29.0	49.6	21.5	1727 (60.1)	15.4	51.6	33.0	2015 (58.1)	16.3	60.5	23.2
**Had previous child loss**	***P*** **≤ 0.05**				***P*** **≤ 0.001**				***P*** **≤ 0.001**			
No	1085 (74.5)	24.5	49.5	26.0	2186 (76.1)	11.9	48.9	39.2	2849 (82.2)	14.1	57.9	28.0
Yes	371 (25.5)	33.2	48.4	18.3	687 (23.9)	18.0	5.4	28.7	617 (17.8)	19.0	62.1	18.9
**Health Insurance**					***P*** **≤ 0.001**				***P*** **≤ 0.001**			
Uninsured	–	–	–	–	773 (26.9)	22.3	47.9	29.8	1311 (37.8)	19.6	58.2	22.3
Insured	–	–	–	–	2100 (73.1)	10.1	50.7	39.2	2155 (62.2)	12.2	58.9	28.9
**Household wealth**	***P*** **≤ 0.001**				***P*** **≤ 0.001**							
Poorest	335 (23.0)	39.9	49.7	10.4	637 (22.2)	25.7	61.2	13.1	747 (21.6)	24.1	59.7	16.2
Poorer	347 (23.9)	31.6	53.5	14.9	621 (21.6)	20.8	51.0	28.2	694 (20.0)	18.5	64.3	17.2
Middle	277 (19.0)	31.0	51.2	17.7	568 (19.8)	8.3	56.6	35.1	676 (19.5)	17.7	60.7	21.6
Richer	286 (19.6)	17.1	49.2	33.7	517 (18.0)	7.0	45.0	48.0	709 (20.5)	8.7	57.9	33.4
Richest	211 (14.5)	4.9	39.0	56.1	530 (18.5)	1.5	50.0	36.6	640 (18.5)	4.7	49.8	45.5
**Urban-Rural residence**	***P*** **≤ 0.001**				***P*** **≤ 0.001**				***P*** **≤ 0.001**			
Urban	498 (34.2)	14.5	47.4	38.0	1214 (42.3)	5.9	44.2	49.9	1464 (42.3)	9.7	54.0	36.3
Rural	958 (65.8)	33.0	50.2	16.8	1659 (57.8)	18.8	54.2	27.0	2002 (57.8)	18.8	62.0	19.2
**Region of residence**	***P*** **≤ 0.001**				***P*** **≤ 0.001**				***P*** **≤ 0.001**			
Western	154 (10.6)	29.7	48.3	21.9	306 (10.7)	21.5	44.8	33.8	400 (11.5)	12.4	43.5	44.1
Central	112 (7.7)	33.0	45.6	21.4	279 (9.7)	12.0	54.8	33.2	341 (9.8)	14.8	56.8	28.4
Greater Accra	177 (12.2)	15.8	36.4	47.9	451 (15.7)	8.1	27.7	64.3	332 (9.6)	9.8	46.4	44.0
Volta	103 (7.1)	38.6	51.1	10.3	214 (7.5)	17.4	57.8	24.8	285 (8.2)	25.5	59.0	16.5
Eastern	195 (13.4)	40.7	44.7	14.6	327 (11.4)	6.8	55.8	37.3	402 (11.6)	19.3	58.3	22.3
Ashanti	222 (15.2)	17.0	50.4	32.6	511 (17.8)	9.0	44.1	46.9	788 (22.7)	12.9	65.9	21.3
Brong Ahafo	115 (7.9)	24.2	60.1	15.8	258 (9.0)	16.1	62.4	21.5	330 (9.5)	14.5	60.6	25.0
Northern	278 (19.1)	27.7	53.6	18.7	321 (11.2)	24.9	56.1	19.1	388 (11.2)	17.7	66.4	16.0
Upper East	61 (4.2)	13.8	53.1	33.1	120 (4.2)	11.2	71.0	17.7	112 (3.3)	4.6	64.2	31.3
Upper West	40 (2.7)	20.5	63.2	16.3	85 (3.0)	9.7	74.2	16.1	88 (2.6)	15.2	70.8	14.1

#### Data preparation and Analysis

Data analyses began by cleaning and recoding variables of interest in STATA version 14. The GMICS predefined survey weights for the differential probability selection of sample were accounted for with the Taylor linearization technique [24, 25]. This procedure adjusted for the clustering, stratification, and design effects within the datasets. Univariable analyses were initially performed on all three waves of datasets by calculating frequencies and percentages of all the variables (see Table 1 - second, sixth, and tenth columns). Secondly, simple Poisson regression was used to determine whether there was a significant trend in ANC visits over the three data waves (2006, 2011, 2018) (Additional file 1). Furthermore, bivariable analyses were performed with a chi-square test of independence to estimate the relationship between the explanatory variables and the outcome variable as presented in Table 1. Lastly, multivariable analyses with robust multinomial logistic regression models were conducted, treating the “less than 4 visits” category in the outcome variable (antenatal care attendance) as the base. All the explanatory variables were independently (Table 2) and simultaneously (see Table 3) regressed onto the outcome variable, regardless of the statistical significance value in the bivariable analyses. The same processes were repeated for all the three datasets used in this study, setting the significance alpha level at 0.05. The relative risk ratio and the adjusted relative risk ratio were reported.

**Table 2:** Unadjusted multinomial logit model showing correlates of ANC visits in Ghana from 2006 to 2017-2018

	2006			2011			2017-2018		
	< 4	4-7	8+	< 4	4-7	8+	< 4	4-7	8+
**Age (years)**	**Base**	**RR [95% CI]**	**RR [95% CI]**	**Base**	**RR [95% CI]**	**RR [95% CI]**	**Base**	**RR [95% CI]**	**RR [95% CI]**
15-24	1	Ref.	Ref.	1	Ref.	Ref.	1	Ref.	Ref.
25-34	1	1.3 [0.9, 1.8]	1.9** [1.3, 2.8]	1	1.5* [1.1, 2.2]	2.1*** [1.4, 3.2]	1	1.2 [0.9, 1.6]	1.7** [1.2, 2.5]
35+	1	1.2 [0.8, 1.7]	1.5 [0.9, 2.3]	1	1.2 [0.8, 1.7]	1.1 [0.7, 1.8]	1	1.2 [0.8, 1.7]	1.3 [0.9, 2.0]
**Education level**									
None or pre-primary	1	Ref.	Ref.	1	Ref.	Ref.	1	Ref.	Ref.
Primary	1	1.0 [0.7, 1.5]	1.1 [0.7, 1.8]	1	1.2 [0.8, 1.8]	2.7*** [1.8, 4.2]	1	0.852 [0.6, 1.2]	1.298 [0.8, 2.1]
JHS	1	2.2*** [1.5, 3.2]	3.8*** [2.5, 5.9]	1	1.9** [1.3, 3.0]	5.8*** [3.7, 9.2]	1	1.6** [1.1, 2.1]	2.8*** [1.8, 4.2]
Secondary & above	1	4.0** [1.5,11.01]	17.7*** [6.6, 47.4]	1	4.6*** [2.0, 10.6]	25.3*** [11.1, 57.5]	1	2.0** [1.3, 3.3]	7.9*** [4.5, 13.6]
**Polygyny**									
Never/formerly married	1	Ref.	Ref.	1	Ref.	Ref.	1	Ref.	Ref.
In one union	1	1.4 [0.9, 2.2]	2.0* [1.1, 3.6]	1	1.7* [1.0, 2.8]	1.5 [0.9, 2.5]	1	1.6** [1.2, 2.2]	1.8** [1.2, 2.6]
Have co-wives	1	1.5 [0.9, 2.4]	1.1 [0.6, 2.0]	1	0.8 [0.5, 1.4]	0.3*** [0.2, 0.6]	1	1.3 [0.9, 1.9]	0.7 [0.4, 1.3]
**Wanted the last child**									
Yes	1	Ref.	Ref.	1	Ref.	Ref.	1	Ref.	Ref.
Later/No More/others	1	0.5*** [0.4, 0.6]	0.4*** [0.2, 0.5]	1	0.7* [0.6, 0.9]	0.6** [0.4, 0.8]	1	0.7* [0.5, 0.9]	0.5*** [0.4, 0.8]
**Parity**									
Primiparous	1	1.0 [0.7, 1.5]	1.3 [0.9, 2.0]	1	2.0** [1.3, 3.0]	2.9*** [1.9, 4.5]	1	1.1 [0.8, 1.5]	1.7** [1.2, 2.4]
Double	1	1.4 [1.0, 2.1]	1.9** [1.2, 3.0]	1	1.0 [0.6, 1.6]	1.3 [0.8, 2.0]	1	1.2 [0.9, 1.8]	1.6* [1.1, 2.4]
Multiparous	1	Ref.	Ref.	1	Ref.	Ref.	1	Ref.	Ref.
**Had previous child loss**									
No	1	Ref.	Ref.	1	Ref.	Ref.	1	Ref.	Ref.
Yes	1	0.7* [0.5, 1.0]	0.5*** [0.4, 0.8]	1	0.7 [0.5, 1.0]	0.5*** [0.3, 0.7]	1	0.8 [0.6, 1.1]	0.5*** [0.3, 0.7]
**Health Insurance**									
Uninsured	1	–	–	1	0.4*** [0.3, 0.6]	0.3*** [0.2, 0.5]	1	0.6*** [0.5, 0.8]	0.5*** [0.4, 0.7]
Insured	1	Ref.	Ref.	1	Ref.	Ref.	1	Ref.	Ref.
**Household wealth**									
Poorest	1	Ref.	Ref.	1	Ref.	Ref.	1	Ref.	Ref.
Poorer	1	1.4 [0.9, 2.0]	1.8* [1.1, 3.0]	1	1.0 [0.7, 1.6]	2.7*** [1.6, 4.3]	1	1.4 [1.0,2.0]	1.4 [0.9, 2.1]
Middle	1	1.3 [0.9, 2.0]	2.2* [1.2, 4.1]	1	2.9*** [1.5, 5.3]	8.3*** [4.1, 16.5]	1	1.4 [0.9, 2.1]	1.8* [1.1, 2.9]
Richer	1	2.3** [1.4, 3.8]	7.6*** [4.2, 13.6]	1	2.7*** [1.5, 4.8]	13.4*** [7.6, 23.9]	1	2.7*** [1.7, 4.3]	5.7*** [3.5, 9.3]
Richest	1	6.4*** [2.7, 14.8]	43.7*** [17.7, 108.0]	1	9.1*** [3.8, 21.7]	84.0*** [34.6, 203.8]	1	4.3*** [2.2, 8.4]	14.6*** [7.6, 27.7]
**Urban-Rural residence**									
Urban	1	Ref.	Ref.	1	Ref.	Ref.	1	Ref.	Ref.
Rural	1	0.5*** [0.3, 0.7]	0.2*** [0.1, 0.3]	1	0.4*** [0.2, 0.6]	0.2*** [0.1, 0.3]	1	0.6** [0.4, 0.8]	0.3*** [0.2, 0.4]
**Region of residence**									
Western	1	0.7 [0.3, 1.5]	0.2** [0.1, 0.6]	1	0.6 [0.2, 2.2]	0.2** [0.1, 0.6]	1	0.7 [0.4, 1.5]	0.8 [0.4, 1.5]
Central	1	0.6 [0.3, 1.3]	0.2** [0.1, 0.6]	1	1.3 [0.4, 4.8]	0.3 [0.1, 1.1]	1	0.8 [0.4, 1.6]	0.4* [0.2, 0.9]
Greater Accra	1	Ref.	Ref.	1	Ref.	Ref.	1	Ref.	Ref.
Volta	1	0.6 [0.3, 1.2]	0.1*** [0.0, 0.2]	1	1.0 [0.3, 3.7]	0.2** [0.0, 0.7]	1	0.5 [0.2, 1.0]	0.1*** [0.1, 0.3]
Eastern	1	0.5* [0.2, 1.0]	0.1*** [0.1, 0.3]	1	2.4 [0.5, 10.8]	0.7 [0.2, 2.7]	1	0.6 [0.3, 1.3]	0.3*** [0.1, 0.5]
Ashanti	1	1.3 [0.6, 2.9]	0.6 [0.3, 1.4]	1	1.4 [0.4, 5.6]	0.7 [0.2, 2.3]	1	1.1 [0.5, 2.2]	0.4** [0.2, 0.8]
Brong Ahafo	1	1.1 [0.5, 2.5]	0.2** [0.1, 0.6]	1	1.1 [0.3, 4.4]	0.2** [0.0, 0.6]	1	0.9 [0.4, 1.8]	0.4* [0.182, 0.8]
Northern	1	0.8 [0.4, 1.7]	0.2** [0.1, 0.6]	1	0.7 [0.2, 2.3]	0.1*** [0.0, 0.3]	1	0.8 [0.4, 1.5]	0.2*** [0.1, 0.4]
Upper East	1	1.7 [0.7, 3.9]	0.8 [0.3, 2.0]	1	1.8 [0.5, 7.0]	0.2** [0.1, 0.6]	1	3.0* [1.3, 6.9]	1.5 [0.7, 3.5]
Upper West	1	1.3 [0.7, 2.7]	0.34** [0.1, 0.6]	1	2.2 [0.6, 8.0]	0.2** [0.1, 0.7]	1	1.0 [0.5, 1.9]	0.2*** [0.1,0.4]

**Table 3:** Adjusted multinomial logit model displaying correlates of ANC visits in Ghana from 2006 to 2017-2018

	2006			2011			2017-2018		
	< 4	4-7	8+	< 4	4-7	8+	< 4	4-7	8+
**Age (years)**	**Base**	**ARR [95% CI]**	**ARR [95% CI]**	**Base**	**ARR [95% CI]**	**ARR [95% CI]**	**Base**	**ARR [95% CI]**	**ARR [95% CI]**
15-24	1	Ref.	Ref.	1	Ref.	Ref.	1	Ref.	Ref.
25-34	1	1.1 [0.7, 1.6]	1.3 [0.7, 2.3]	1	2.1** [1.3, 3.4]	2.7** [1.5,4.9]	1	1.0 [0.7, 1.5]	1.5 [0.9, 2.5]
35+	1	1.3 [0.8, 2.1]	1.9 [1.0, 3.8]	1	2.1** [1.2, 3.6]	2.5** [1.3, 5.1]	1	1.1 [0.7, 1.9]	1.7 [0.9, 3.0]
**Education level**									
None or pre-primary	1	Ref.	Ref.	1	Ref.	Ref.	1	Ref.	Ref.
Primary	1	1.5* [1.0,2.4]	1.5 [0.9, 2.6]	1	1.043 [0.7, 1.6]	1.6 [0.9, 2.6]	1	0.9 [0.7, 1.3]	1.2 [0.7, 2.1]
JHS	1	3.1*** [2.0, 4.7]	3.5*** [2.1, 6.0]	1	1.3 [0.8, 2.1]	2.0* [1.1, 3.4]	1	1.5 [1.0, 2.2]	1.8* [1.1, 3.1]
Secondary & above	1	4.1** [1.4, 12.0]	8.0*** [2.6, 24.5]	1	1.3 [0.5, 3.5]	2.2 [0.8, 6.0]	1	1.2 [0.7, 2.2]	2.3* [1.2, 4.5]
**Polygyny**									
Never/formerly married	1	Ref.	Ref.	1	Ref.	Ref.	1	Ref.	Ref.
In one union	1	1.2 [0.7, 2.1]	1.7 [0.9,3.4]	1	2.1* [1.1, 3.9]	1.3 [0.7, 2.6]	1	1.4 [1.0, 2.1]	1.3 [0.8, 2.1]
Have co-wives	1	1.5 [0.8, 2.8]	1.3 [0.6, 3.0]	1	1.3 [0.6, 2.5]	0.6 [0.3, 1.2]	1	1.3 [0.8, 2.1]	0.9 [0.5, 1.6]
**Wanted the last child**									
Yes	1	Ref.	Ref.	1	Ref.	Ref.	1	Ref.	Ref.
Later/No More/others	1	0.5*** [0.4, 0.6]	0.3*** [0.2, 0.5]	1	0.6*** [0.5, 0.8]	0.4*** [0.3, 0.6]	1	0.8 [0.6, 1.0]	0.646* [0.5, 0.9]
**Parity**									
Primiparous	1	0.9 [0.5, 1.6]	1.2 [0.6, 2.4]	1	3.0*** [1.6, 5.7]	2.9** [1.4, 5.8]	1	1.1 [0.7, 1.8]	1.6 [0.9, 2.9]
Double	1	1.1 [0.7, 1.8]	1.2 [0.7, 2.2]	1	1.2 [0.7, 2.0]	1.0 [0.6, 1.8]	1	1.1 [0.7, 1.7]	1.1 [0.7, 1.8]
Multiparous	1	Ref.	Ref.	1	Ref.	Ref.	1	Ref.	Ref.
**Had previous child loss**									
No	1	Ref.	Ref.	1	Ref.	Ref.	1	Ref.	Ref.
Yes	1	0.8 [0.6, 1.2]	0.7 [0.5, 1.1]	1	0.9 [0.6, 1.4]	0.8 [0.5, 1.4]	1	0.8 [0.5, 1.2]	0.6* [0.4, 1.0]
**Health Insurance**									
Uninsured	1	–	–	1	0.6** [0.4, 0.9]	0.5*** [0.3, 0.7]	1	0.7* [0.5, 0.9]	0.7* [0.5, 0.9]
Insured	1	Ref.	Ref.	1	Ref.	Ref.	1	Ref.	Ref.
**Household wealth**									
Poorest	1	Ref.	Ref.	1	Ref.	Ref.	1	Ref.	Ref.
Poorer	1	1.5 [1.0, 2.3]	2.3** [1.3, 4.2]	1	1.0 [0.6, 1.7]	1.9* [1.1, 3.3]	1	1.6* [1.1, 2.3]	1.3 [0.8, 2.2]
Middle	1	1.3 [0.8, 2.1]	2.4* [1.1, 5.0]	1	2.6** [1.3, 5.2]	4.8*** [2.3, 10.2]	1	1.5 [0.9, 2.5]	1.4 [0.8, 2.5]
Richer	1	1.9 [1.0, 3.6]	6.0*** [2.7, 13.4]	1	2.3* [1.1, 4.8]	5.9*** [2.5, 13.9]	1	2.799*** [1.566,5.002]	4.001*** [2.208,7.251]
Richest	1	3.335* [1.251,8.893]	15.64*** [4.868,50.25]	1	7.3** [2.1, 25.2]	24.3*** [6.7, 87.8]	1	3.8** [1.7, 8.5]	5.9*** [2.6, 13.1]
**Urban-Rural residence**									
Urban	1	Ref.	Ref.	1	Ref.	Ref.	1	Ref.	Ref.
Rural	1	0.6* [0.4, 1.0]	0.7 [0.4, 1.2]	1	0.6 [0.3, 1.2]	0.6 [0.3, 1.1]	1	0.9 [0.6, 1.4]	0.6* [0.4, 1.0]
**Region of residence**									
Western	1	1.3 [0.6, 3.0]	0.8 [0.3, 1.9]	1	1.9 [0.4, 9.4]	0.9 [0.2, 3.7]	1	1.1 [0.5, 2.4]	2.0 [0.9, 4.4]
Central	1	1.3 [0.6, 2.9]	0.9 [0.4, 2.5]	1	3.5 [0.8, 16.1]	1.3 [0.4, 4.9]	1	1.1 [0.6, 2.3]	1.0 [0.4, 2.1]
Greater Accra	1	Ref.	Ref.	1	Ref.	Ref.	1	Ref.	Ref.
Volta	1	1.4 [0.6, 3.3]	0.5 [0.2, 1.4]	1	3.1 [0.7, 14.6]	1.1 [0.3, 4.3]	1	0.9 [0.4, 1.9]	0.6 [0.2, 1.3]
Eastern	1	0.9 [0.4, 2.0]	0.5 [0.2, 1.0]	1	5.7* [1.1, 29.6]	2.2 [0.5, 9.0]	1	1.0 [0.5, 1.9]	0.6 [0.3, 1.3]
Ashanti	1	2.364 [1.0, 5.6]	1.9 [0.8, 4.3]	1	3.8 [0.8, 18.7]	2.7 [0.6, 11.2]	1	1.4 [0.6, 2.8]	0.6 [0.3, 1.3]
Brong Ahafo	1	2.2 [0.9, 5.4]	0.9 [0.3, 2.8]	1	3.7 [0.8, 17.8]	0.9 [0.2, 3.8]	1	1.4 [0.7, 2.9]	1.0 [0.5, 2.3]
Northern	1	2.7* [1.2, 6.3]	1.8 [0.7, 4.8]	1	2.6 [0.6, 12.1]	1.0 [0.3, 3.9]	1	1.7 [0.8, 3.6]	1.1 [0.5, 2.6]
Upper East	1	6.4*** [2.5, 16.4]	9.7*** [3.6, 26.5]	1	7.7* [1.6, 37.5]	2.4 [0.6, 9.6]	1	6.6*** [2.5, 17.1]	7.3*** [2.6, 20.5]
Upper West	1	5.8*** [2.6, 12.6]	3.4* [1.2, 9.5]	1	8.3** [1.8, 39.1]	2.0 [0.5, 7.9]	1	2.1 [1.0, 4.5]	0.9 [0.4, 2.2]
**Model details**									
Number of strata	20			20			20		
Number of Primary Sampling Unit	291			775			649		
Number of Observations	1456			2873			3466		

Exponentiated coefficients; 95% confidence intervals in brackets. * *p* < 0.05, ** *p* < 0.01, *** *p* < 0.001.

#### Ethical approval and Data availability

This study was performed following the Declaration of Helsinki and approved by the appropriate ethics committee. The original survey data utilized for this secondary data analysis study was collected by trained field enumerators on behalf of UNICEF and GSS. The MICS team of UNICEF-Ghana, The Ethical Review Board of the Ghana Health Service, and the Ghana Statistical Service approved the study that collected the original survey data. Therefore, ethics approval for this current study was not required since the data is secondary and is available in the public domain. Before the collection of the original survey data, informed consent was obtained from all the respondents. Adult verbal consents and child assents were obtained for the respondents younger than eighteen from their parents/guardians/adult household members to participate in the survey. Additionally, participants were assured of anonymity and confidentiality. More details regarding the data and ethical standards are available at: https://mics.unicef.org/surveys. All methods were performed in accordance with the relevant guidelines and regulations.

## Results

### Maternal socio-demographic characteristics

Overall, 7795 women aged 15 to 49 years from all the three waves who had delivered 2 years before the data collection periods were included in this study. Out of the 1456 mothers in 2006, 47.6% were 25-34 years old. This percentage increased to 50.0% in 2011 but reduced in 2017-2018 (47.2%). Regarding education, majority of the mothers had no or pre-primary education (36.9%) in 2006, however, in 2011 (35.0%) and 2017-2018 (38.7%), the majority were those who had junior high education. Additionally, the percentage of women living in rural areas was higher (65.8%) in 2006 but decreased in 2011 (57.8%) which remained the same in 2017-2018. About 73.1% of mothers in 2011 were insured but that of insured mothers in 2017-2018 was lesser (62.2%). The remaining of the descriptive statistics are shown in Table 1.

### Trends in antenatal care visits in Ghana from 2006 to 2017-2018

There was a statistically significant relationship between years and ANC visits from 2006 through 2018 (Additional file 1). Out of the mothers who received antenatal care during pregnancy in the 2 years preceding the survey in 2006, 26.7% had less than 4 antenatal care visits (undesirable antenatal care). The percentage of those who had undesirable antenatal care drastically reduced in 2o11 (13.4%) and then it slightly increased in 2017-2018 (15.0%). About 49.3% of mothers had adequate antenatal care (4-7 antenatal care visits) in 2006 but there was a slight increase in the percentage of adequate antenatal care among mothers in 2011 (50.0%). The percentage further increased to 58.6% in 2017-2018. Generally, there was a consistent increase in the proportion of women having adequate and optimal ANC attendance from 2006 to 2018 across the women’s socio-demographic segments. However, marked socioeconomic and demographic disparities were observed. For instance, the proportion of women with secondary or higher education [2006, 55.1%; 2011, 60.9%; 2017-2018, 45.2%] consistently had at least three times higher proportion of optimal ANC attendance compared to women without education/pre-primary education [2006, 16.1%; 2011, 18.4%; 2017-2018, 15.8%]. Also, women in the richest households [2006, 56.1%; 2011, 36.6%; 2017-2018, 45.5%] had at least three times higher proportion of optimal ANC attendance compared to women of the poorest households [2006, 10.4%; 2011, 13.1%; 2017-2018, 16.2%]. The proportion of urban women who had optimal ANC attendance [2006, 38.0%; 2011, 49.9%; 2017-2018, 36.3%] for their recent child was consistently at least twice as high compared to rural women [2006, 16.8%; 2011, 27.0%; 2017-2018, 19.2%]. The results showed a statistically significant relationship between all the explanatory variables and antenatal care visits in 2006, 2011 and 2017-2018 as shown in Table 1.

### Correlates of antenatal care visits in Ghana from 2006 to 2017-2018

Tables 2 and 3 represent the unadjusted and adjusted multinomial logit models respectively, showing the correlates of ANC visits in Ghana from 2006 to 2017-2018. In the adjusted multinomial logit model, generally, upward attainment of formal education, health insurance coverage, increasing household wealth, and residing in the Upper East region were consistently associated with a higher likelihood of adequate and/or optimal ANC attendance from 2006 through 2017-2018 relative to undesirable ANC attendance among childbearing women in Ghana. Examples of some of the consistent factors in the 2017-2018 adjusted model are interpreted for purposes of brevity result interpretation. For the most recent year, compared to women with no formal education, women who had attained a junior high school education (adjusted relative risk ratio [ARR] = 1.8, 95% CI: 1.1, 3.1) or secondary education and above (ARR = 2.3, 95% CI: 1.2, 4.5) were associated with a higher likelihood of optimal ANC attendance. Unexpectedly, mothers who had children from unplanned pregnancies were consistently associated with a lower likelihood of adequate and/or optimal ANC attendance from 2006 through 2017-2018 relative to undesirable ANC attendance. For instance, compared to women who wanted their recent child, women who had unplanned births (ARR = 0.6, 95% CI: 0.5, 0.9) were negatively associated with optimal ANC attendance relative to undesirable ANC attendance. Although death of previous child was only significantly related to ANC attendance in the 2017-2018 model, it is worth mentioning because of the unexpected observed association. That is, we unexpectedly observed that women who had lost a previous child to death (ARR = 0.6, 95% CI: 0.4, 1.0) was associated with a lower likelihood of optimal ANC attendance for their recent child. Parity was also observed to be significantly related to ANC attendance in the 2011 model. Lastly, women residing in the Upper East Region were consistently found to have a higher likelihood of adequate and optimal ANC attendance with increased odds of 6.6 (95% CI: 2.5, 17.1) and 7.3 (95% CI: 2.6,20.5) respectively in 2017-2018.

## Discussion

The World Health Organization (WHO) recommends antenatal visits of at least eight times during pregnancy and to initiate antenatal care (ANC) in the first trimester [13]. However, many women in developing countries do not adhere to this recommendation [26]. In this study, we examined the trends of, and factors associated with ANC utilization in Ghana. We found that the factors associated with adequate or optimal ANC attendance from 2006 through 2017-2018 included higher attainment of formal education, health insurance coverage, increasing household wealth, urban residence, and residing in the Upper East Region. Also, there has been an increase in the number of women having adequate ANC attendance from 2006 to 2017-2018; the proportion of women increased from 49.3 to 49.98% from 2006 to 2011 and 58.61% in 2017-2018.

The proportion of mothers achieving adequate ANC (4-7 ANC visits) increased from 49.3% in 2006 to 49.98% in 2011 to 58.61% in 2017-2018. Consistent with this study, Alhassan and colleagues found that there has been an increase in the trends of ANC service utilization in Ghana in the past decade [27]. This may be due to the enactment and implementation of the Ghana National Health Insurance Scheme (NHIS) in 2003 which aimed to offer free medical care to pregnant women. This policy includes the provision of a comprehensive exemption package that provides ANC, postnatal care, and skilled services [28]. The rise in utilization of ANC services may also be due to a change in the WHO recommendations on ANC. In 2003, WHO recommended that in low-income countries pregnant women without any complications were required to visit health facilities at least four times during their pregnancies [29]. However, this policy was reviewed by the WHO in 2016, recommending at least eight ANC visits throughout pregnancy to reduce perinatal deaths irrespective of the income level [13]. This increase in the minimum recommended number of ANC visits means that pregnant women would have to visit healthcare facilities more frequently than they used to do before 2016.

From this study, an increase in the level of education was found to be associated with adequate ANC attendance. Women who had secondary education and higher or junior high school were associated with adequate ANC attendance compared to women with no formal education. Similar findings have been observed in many studies in low and middle-income countries [30–32]. Higher education would increase women’s awareness, knowledge of ANC services utilization and its consequences, thereby informing their healthcare decision making. Higher education is associated with an increase in the knowledge of obstetric complications, thereby leading to improved utilization of ANC services [33]. The positive effect of the level of education complimented by the introduction of free maternal care policy on Ghana’s NHIS from 2008 may have particularly contributed to the increase in proportion of higher ANC visits in 2011 and 2017-2018.

Unplanned pregnancy was negatively associated with ANC attendance. This finding is consistent with previous studies in Ghana [34] and Bangladesh [35, 36] that found that women who reported unintended pregnancies at conception and did not terminate their pregnancies were at higher risk of not using ANC services. Therefore, it is imperative to motivate and incorporate women with unplanned pregnancies in the mainstream healthcare service policies to increase their ANC attendance and to lessen the related undesirable outcomes [35]. Although it was only significantly related to ANC attendance in the 2017-2018 model, it is worth mentioning due to its unexpected observed association that, the death of a previous child was negatively associated with ANC attendance. This concurs with a study in Nigeria that found the death of a preceding child to be associated with a lower likelihood of ANC attendance in the univariate models [37]. Given that poor healthcare-seeking behaviour and health outcomes have been linked to low socioeconomic status among people including pregnant women [38, 39], covering the cost of medical care for pregnant women under the NHIS could account for the increased ANC attendance. From our study, health insurance coverage was positively associated with ANC attendance. This concurs with multiple previous studies conducted in Ghana that found the possession of national health insurance to be positively associated with attending ANC at least four times [18, 20, 34, 40, 41]. Therefore, the Government of Ghana should continually strengthen the NHIS and ensure that its goal of universal coverage is achieved given that it has the potential to help Ghana meet the SDG 3 target by 2030.

In this study, the likelihood of optimal utilization of ANC visits was found to be associated with an increase in household wealth; women from the richest homes were more likely to obtain optimal ANC services compared to women from the poorest households in all the three survey waves. Economic barriers, particularly the inability to pay have been linked to decreased utilization of ANC services as observed by studies conducted in Nigeria and Nepal [42, 43]. These factors influence women’s ability to regulate their health and facilitate easy access to maternal health care. Using a nationally representative data, the Ghana Statistical Service [44] reported that women in the middle and highest wealth quintiles were more likely to receive ANC services from healthcare professionals than those in the lowest quintiles. Despite the fee exemptions for ANC services, poor women are still unlikely to utilize these services due to lack of adequate information, other costs (of drugs and supplies), transportation costs, and discrimination by some health professionals [45].

We found that women residing in urban areas were more likely to attend at least eight ANC services during pregnancy, compared to women residing in rural areas. This aligns with the findings of studies conducted in Ghana and other developing countries [46–49]. Proximity to health facilities, bad road networks, traditional beliefs against patronage of health services, and unfavourable working conditions in rural areas for health professionals have been cited as some of the reasons for the low utilization of ANC services in most developing countries [47, 50]. Also, compared to women residing in other regions, women residing in the Upper East Region of Ghana has the highest likelihood of optimal ANC service utilization. This finding is in line with that of Sakeah and colleagues who found that 35.6% of women residing in Navrongo in the Upper East Region visit ANC clinic adequately compared to women living in Kintampo (33.2%) and Dodowa (33.7%) in the erstwhile Brong Ahafo and Greater Accra Regions, respectively [51]. They also observed that rural areas in the Upper East Region have more community health-based planning services (CHPS) compounds than most rural areas in other regions in Ghana [51].

Further to the above, we observed that parity was significantly associated with ANC attendance only in the 2011 model: primiparous women were more likely to utilise ANC services. Congruent with this finding in the 2011 logit model, previous studies [30, 52, 53] have also found that women with higher parity tend to use ANC services less. Potentially, this finding may be related to the fact that multiparous women may have pregnancy-related lived experiences that enable them to handle issues on their own with limited reliance of ANC services. Primiparous women may lack these experiences which may serve as a push factor to utilise ANC services. However, the effect of parity on ANC visit was not significant in the model for the recent data wave.

The findings of this study provide several implications for policy and practice. Mothers who could not obtain optimal ANC services stand the chance of having complications associated with their pregnancies or childbirths. These findings provide empirical-based evidence to improve ANC services utilization in the regions where women find it difficult to have at least eight ANC visits. Deliberate policies aimed at reducing poverty, promoting maternal and girl-child education, and improving general livelihood in rural settings should be enacted as these policies have the potential of improving the health-seeking behaviours of women in rural areas to help reduce maternal and child deaths. Moreover, information, education, and communication aimed at stimulating health-seeking behaviours should be promoted at all levels to increase the use of antenatal care services and lower the prevalence of adverse pregnancy outcomes. We also recommend the identification and harnessing of community-level structures to improve maternal health in rural areas [54], which includes ensuring collaboration between traditional birth attendants (TBAs) and health professionals in delivering essential maternal health services. A typical example is delegating community members designated as mother-to-mother support group leaders or TBAs to serve as targeted support systems for pregnant mothers to help their enrolment and retention in ANC services. This will ensure the early utilization of ANC services which in turn increase the chances of meeting the minimum target of eight ANC visits. Furthermore, health insurance coverage should be expanded to cover all costs related to pregnancy and childbirth, whiles reducing unnecessary payments during ANC visits. There should also be the training of more healthcare providers and the initiation of targeted motivation schemes to attract and retain qualified staff in deprived communities to meet the growing shortages, especially in rural areas. The Government of Ghana, through the Ministry of Health and Ghana Health Service, should in the long term provide adequate infrastructure (such as hospitals, clinics, health centres, laboratories, essential equipment, and motorable roads) and ensure that they are functioning efficiently. In the short and medium terms, understaffed CHPS compounds, clinics, and health centres in rural areas must collaborate with adequately staffed hospitals to ensure proper referral systems and utilize the expertise of their staff through telehealth and telemedicine. These findings should inform policy decisions at all levels and engender further epidemiological inquiries through mixed method designs to unravel community and facility level oddities on the factors influencing ANC utilization.

A key strength of this study was the use of a large, nationally representative survey datasets collected in three waves by the Ghana Multiple Indicator Cluster Survey (GMICS) in 2006, 2011 and 2017-2018 based on a standardised methodology for analyses. Therefore, our findings can be generalized. Secondly, the study employed a complex sample analytic design to account for sampling units and weighting. In addition, the study unravelled the population of pregnant women who are more likely to achieve the optimal ANC visits as recommended by WHO, the predictive sociodemographic factors and social inequalities and the achieved progress. The main limitation of the study is that we used secondary data which utilized a cross-sectional design. Hence, the associations observed in this study did not infer a causal relationship between the predictors and the outcome variables. The study was also restricted to variables available in the GMICS Data.

## Conclusions

This study has offered insights into the trends of ANC utilization and its associated factors in Ghana to inform policy and practice. This study’s analyses showed a consistent increase in the proportion of women having adequate and optimal ANC attendance from 2006 to 2017-2018. The proportion of mothers achieving adequate ANC (4-7 ANC visits) increased from 49.3% in 2006 to 49.98% in 2011 to 58.61% in 2017-2018. In the multivariable model, women with upward attainment of formal education, health insurance coverage, increasing household wealth, and residing in the Upper East Region of Ghana were consistently associated with a higher likelihood of adequate and/or optimal ANC attendance from 2006 to 2017-2018. Women who are less likely to achieve optimal ANC visits should be targeted by policies towards reducing maternal mortalities and other birth complications. Poverty-reduction policies, promoting maternal and girl-child education, improving general livelihood in rural settings, expanding health insurance coverage and infrastructural access, harnessing community-level structures, and innovative measures such as telehealth and telemedicine are required to increase ANC utilization.

## Supplementary Information

**Additional file 1.** Year of data collection regressed on antenatal care visit and predicted margins.

## Data Availability

The datasets that were used in this study is freely available at https://mics.unicef.org/visitors/sign-in once permission is sought and granted by UNICEF.
