# Supplementary material for: Trends in antenatal care visits and associated factors in Ghana from 2006 to 2018
Source: BMC Pregnancy Childbirth. 2022 Jan 22;22:59. doi: 10.1186/s12884-022-04404-9 (PMC8783507; doi:10.1186/s12884-022-04404-9)
Supplement: Supplementary file 1 — Additional file 1. Year of data collection regressed on antenatal care visit and predicted margins. [file 12884_2022_4404_MOESM1_ESM.docx]

Additional File 1: Year of data collection regressed on antenatal care visit and predicted margins

S Table 1: Data wave (year) regressed onto ANC visits

|  |  | Linearized |  |  |  |  |
| --- | --- | --- | --- | --- | --- | --- |
| ANC Visits | Coef. | Std. Err. | t | P>t | [95% Confidence Interval] | |
|  |  |  |  |  |  |  |
| YEARS |  |  |  |  |  |  |
| 2006 | 0 | (base) |  |  |  |  |
| 2011 | 0.17 | 0.03 | 5.55 | 0.000 | 0.11 | 0.23 |
| 2017 | 0.10 | 0.03 | 3.65 | 0.000 | 0.05 | 0.16 |
|  |  |  |  |  |  |  |
| Intercept | 1.71 | 0.02 | 69.12 | 0.000 | 1.66 | 1.76 |

S Table 2: Adjusted predictions of years with 95% Confidence Interval

|  |  | Delta-method |  |  |  |  |
| --- | --- | --- | --- | --- | --- | --- |
|  | Margin | Std. Err. | t | P>t | [95% Confidence Interval] | |
|  |  |  |  |  |  |  |
| YEARS |  |  |  |  |  |  |
| 2006 | 5.55 | 0.14 | 40.35 | 0.000 | 5.28 | 5.81 |
| 2011 | 6.58 | 0.11 | 60.97 | 0.000 | 6.37 | 6.79 |
| 2017 | 6.15 | 0.08 | 76.49 | 0.000 | 5.99 | 6.31 |
